# Nephroprotective Effects of Polydatin against Ischemia/Reperfusion Injury: A Role for the PI3K/Akt Signal Pathway

**DOI:** 10.1155/2015/362158

**Published:** 2015-10-20

**Authors:** Hong-Bao Liu, Qiu-Hong Meng, Chen Huang, Jian-Bo Wang, Xiao-Wei Liu

**Affiliations:** ^1^Department of Nephrology, Xijing Hospital, Fourth Military Medical University, Xi'an 710032, China; ^2^State Key Laboratory of Cancer Biology, Department of Medical Genetics and Developmental Biology, Fourth Military Medical University, Xi'an 710032, China; ^3^Institute of Materia Medica, School of Pharmacy, Fourth Military Medical University, Xi'an 710032, China

## Abstract

Oxidative stress and inflammation are involved in the pathogenesis in renal ischemia/reperfusion (I/R) injury. It has been demonstrated that polydatin processed the antioxidative, anti-inflammatory, and nephroprotective properties. However, whether it has beneficial effects and the possible mechanisms on renal I/R injury remain unclear. In our present study I/R models were simulated both *in vitro* and *in vivo*. Compared with vehicle control, the administration of polydatin significantly improved the renal function, accelerated the mitogenic response and reduced cell apoptosis in renal I/R injury models, strongly suppressed the I/R-induced upregulation of the expression of tumor necrosis factor-*α*, interleukin-1*β*, cyclooxygenase-2, inducible nitric oxide synthase, prostaglandin E-2, and nitric oxide levels, and dramatically decreased contents of malondialdehyde, but it increased the activity of superoxide dismutase, glutathione transferase, glutathione peroxidase and catalase, and the level of glutathione. Further investigation showed that polydatin upregulated the phosphorylation of Akt in kidneys of I/R injury dose-dependently. However, all beneficial effects of polydatin mentioned above were counteracted when we inhibited PI3K/Akt pathway with its specific inhibitor, wortmannin. Taken together, the present findings provide the first evidence demonstrating that PD exhibited prominent nephroprotective effects against renal I/R injury by antioxidative stress and inflammation through PI3-K/Akt-dependent molecular mechanisms.

## 1. Introduction

Renal ischemia/reperfusion (I/R) injury still remains to be a major medical problem due to the lack of more effective treatment [1, 2]. Inflammatory response and oxidative stress are involved in the pathogenesis in I/R injury [3]. It is likely to be an important therapeutical strategy to implement antioxidant and anti-inflammatory agents to treat renal diseases after I/R injury.

**Figure 1:**
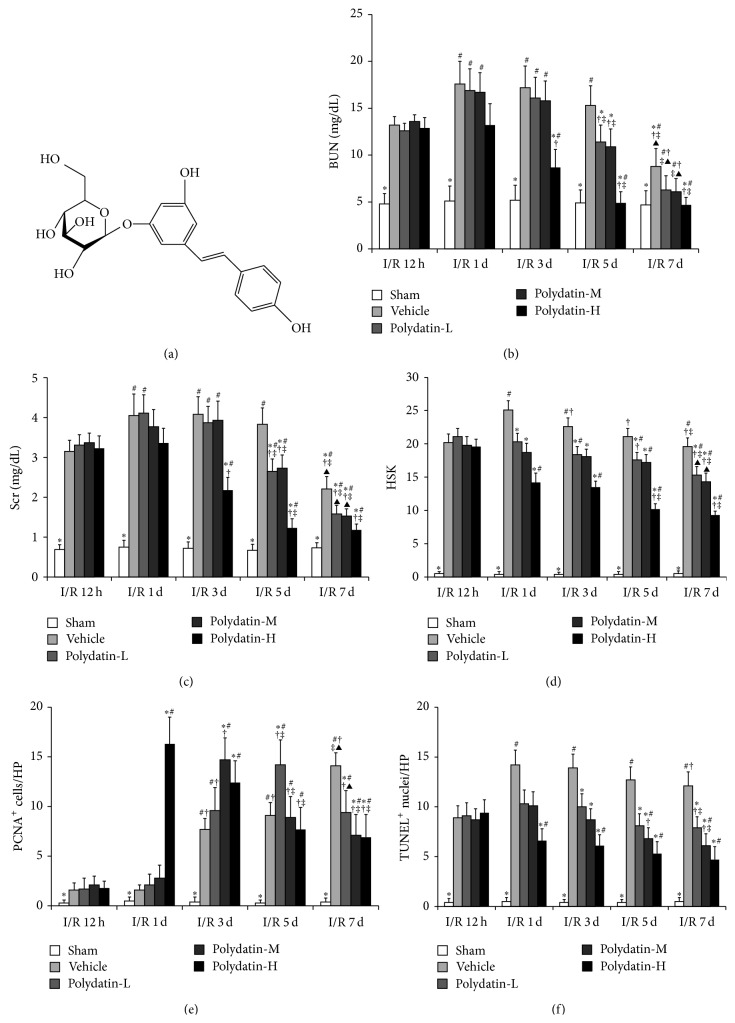
Therapeutical effects of polydatin on renal I/R injury. (a) Chemical structure of polydatin; (b) blood urea nitrogen (BUN); (c) serum creatinine (Scr) in renal I/R mice that received polydatin-L (10 mg/kg), polydatin-M (20 mg/kg), polydatin-H (40 mg/kg), or vehicle (saline with 1% DMSO); (d) histological score of kidney injuries (HSK); (e) immunohistochemical staining for PCNA; and (f) TUNEL in I/R mice that received polydatin-L, polydatin-M, polydatin-H, or vehicle. ^*^ *P* < 0.05 versus vehicle; ^#^ *P* < 0.05 versus I/R 12 h respective group; ^†^ *P* < 0.05 versus I/R 1 d respective group; ^‡^ *P* < 0.05 versus I/R 3 d respective group. ^▲^ *P* < 0.05 versus I/R 5 d respective group.

Polydatin (C_20_H_22_O_8_, resveratrol glucoside with a 3,4′,5-trihydroxystibene-3-*β*-mono-D-glucoside molecular structure, has also been named piceid (Figure 1(a)) is a natural stilbene compound extracted from the dried roots of the perennial herb* Polygonum cuspidatum* Sieb. et Zucc., which has been widely used in traditional Chinese medicine for its multiple pharmacological activities, including its strong antioxidative effects, anti-inflammatory reactions, and improvement of microcirculation [4]. Mounting studies thus far have focused on the beneficial effects of polydatin in prevention of I/R-induced oxidative stress and inflammation. Our previous studies have demonstrated that polydatin exerts cardioprotective effects by activating protein kinase C and mito K_ATP_-dependent signaling and the direct antioxidative stress mechanisms in myocardial I/R rat models [5]. In recent years, polydatin has been suggested to have the properties of nephroprotective effects in diabetes and urate nephropathy [6–9]. However, little work has been done on its underlying possible mechanism as a drug in treating I/R-induced renal dysfunction.

The phosphatidylinositol 3-kinase (PI3K) family is a group of revolutionary conserved signal transduction molecule, which can activate its downstream signaling protein, serine/threonine kinase Akt (also known as protein kinase B, PKB), to participate in the regulation of cell proliferation, survival, apoptosis, and various biological responses including oxidative stress, inflammation, and chemotaxis [10, 11]. We [12] and other authors [13] reported that I/R can induce the activation of PI3K/Akt, which promotes the proliferation and viability of renal tubular epithelial cells. However, whether PI3K/Akt signaling pathways participate in the mechanism of actions of polydatin in I/R injury models is unclear.

Therefore, in this study we tested the potential protective effects of polydatin on renal I/R injury models both* in vitro* and* in vivo*. And by the intervention with wortmannin, the specific PI3K/Akt inhibitor, we explored the role of PI3K/Akt pathway in regulating the therapeutic effects of polydatin in acute renal I/R injury.

## 2. Materials and Methods

### 2.1. Induction of I/R-AKI

Male BALB/c mice (weighing 20~25 g, 7~9 weeks of age) were obtained from the Experimental Animal Center of the Forth Military Medical University (Xi'an, China) and bred in an experimental animal room of specific-pathogen-free (SPF) grade. The experiment procedures were in line with the National Institutes of Health Guide for Care and Use of Laboratory Animals (NIH publication number 85-23, revised 1985) and with the European Communities Council directive of 24 November 1986 (86/608/EEC). All mice were provided with food and water* ad libitum* in a 12 : 12-h light/dark cycle (lights on at 6 am and off at 6 pm). They were allowed to adapt to new surroundings for at least 5 days prior to any experimentation. All efforts were made by us to minimize the suffering of animals in present study.

For the establishment of renal I/R injury models, operation was performed in BALB/c mice by clamping bilateral renal pedicles for renal ischemia of 30-minute time, followed by releasing clamps to allow blood reperfusion as described previously [14]. Briefly, the bilateral renal pedicles of mice were carefully bluntly dissected and then occluded using nontraumatic vascular clamps. The similar operation was performed on mice in sham group, with the pedicles also dissected but not clamped. Animals were ethically sacrificed at 12 h, 1 d, 3 d, 5 d, and 7 d after renal I/R injury, respectively, and whole blood and kidneys were harvested for further analysis.

The animals were randomly divided into five experimental groups as follows: (1) sham group; (2) vehicle group: I/R injury mice with saline vehicle (dimethyl sulfoxide, DMSO 1%) intraperitoneally injected; (3) polydatin low dose group (polydatin-L group): I/R injury mice with polydatin (Weijia Technology Company, Xi'an, China) of 10 mg/kg intraperitoneally injected; (4) polydatin middle dose group (polydatin-M group): I/R injury mice with polydatin of 20 mg/kg intraperitoneally injected; and (5) polydatin high dose group (polydatin-H group): I/R injury mice with polydatin of 40 mg/kg intraperitoneally injected. For groups 3 to 5, the initial dose of polydatin was given before the incision sutured after I/R completed and then continued with daily injections for 6 days. For evaluated PI3K/Akt pathway* in vivo*, 1 mg/kg of wortmannin (Sigma), the specific inhibitor of PI3K/Akt signaling, was given 30 min before operation, intraperitoneally, and then continued with daily injection for 2 days. In studies* in vivo* polydatin and wortmannin were first dissolved in DMSO (Sigma) and then diluted by physiological saline with a final concentration of 1% (vol/vol) DMSO for intraperitoneal application.

### 2.2. Blood Physiochemical Assays

The whole blood drawn from the heart or the retroocular vein plexus was centrifuged at 4°C, 3000 g, for 10 min to obtain the serum sample. The level of blood urea nitrogen (BUN) and serum creatinine (Scr) was measured by the automatic biochemistry analyzer (Beckman; Fullerton, CA).

### 2.3. Histological Score of Kidney Injuries

Kidney samples were fixed overnight in 10% phosphate-buffered formalin and then embedded in paraffin. Renal sections were next prepared and then subjected to hematoxylin and eosin (H&E) staining to assess the histological injury. Evaluation of histological score of kidney injuries (HSK) was performed by a renal pathologist under blinded conditions. HSK was graded using a 4-point quantitative scale as described previously [15]: 0 represented normal histology; 1 represented mild damage [less than 1/3 of nuclear loss (necrosis) in a tubular cross section]; 2 represented moderate damage [more than 1/3 and less than 2/3 of a tubular cross section shows nuclear loss (necrosis)]; 3 represented severe damage [more than 2/3 of nuclear loss (necrosis) per tubular cross section]. We calculated the total score of per kidney section by adding up all 10 scores with a possible maximum injury score of 30.

### 2.4. Immunohistochemical Staining

The tissue sections were subject to immunohistochemical staining for proliferating cell nuclear antigen (PCNA, a marker of mitogenesis) 12 h, 1 d, 3 d, 5 d, and 7 d after I/R injury. For immunohistochemical staining, we used the rabbit specific horseradish peroxidase-diaminobenzidine (HRP-DAB) detection immunohistochemical kit (ab64261, Abcam). After being deparaffinized, hydrated, and peroxidase-blocked, 4 *μ*m sections of kidneys were incubated overnight at 4°C with a rabbit polyclonal FL-261 antibody (1 : 200, sc-7907, Santa Cruz Biotechnology, Santa Cruz, USA). Then sections were incubated with a biotinylated secondary antibody, goat anti-rabbit IgG (H + L), for 10 min. Control experiments were performed by omitting either the primary or secondary antibody. Then we developed the sections using an enzymatic conversion of the DAB, visualizing the color of specific antibody binding sites to change into brown. After all sections were counterstained with hematoxylin (Sigma), they were cleared and finally coverslipped for observation. We randomly selected 10 sections from the corticomedullary area per kidney, counting the number of positive nuclei in high-power fields (HPF, 620 magnification). Then we calculated the mean number of PCNA-positive cells of each kidney. The tubular cell apoptosis in kidneys after I/R was detected by terminal deoxynucleotidyl transferase dUTP nick end labeling (TUNEL) assay following the manufacturer instructions (In Situ Cell Death Detection Kit; Roche China, Ltd.). Meanwhile, we stained all cell nuclei with DAPI (Sigma, USA). We examined TUNEL-stained sections for screening positive nuclei with a fluorescence microscope and selected 10 random fields in renal cortex and outer medullar area in every kidney and then counted them at 640 magnification.

### 2.5. Western Blot

As previously described [1], routinely, we carried out western blot analyses to detect target protein levels in kidney tissues at 3 days after I/R injury with or without polydatin (10, 20, and 40 mg/kg) and wortmannin (1 mg/kg) treatment and cells with or without OGD/R treatment in presence or absence of polydatin (10, 20, and 40 *μ*M) and wortmannin (1 *μ*M). The primary antibodies included rabbit polyclonal antibodies against phospho-Akt (P-Akt, Ser 473, 1 : 1000, Cell Signaling, Danvers, MA, USA), total Akt (T-Akt, 1 : 1000, Cell Signaling, Danvers, MA, USA), cyclooxygenase-2 (COX-2, 1 : 500, Abcom, USA), inducible nitric oxide synthase (iNOS, 1 : 1000, Abcom, USA), and *β*-actin (1 : 2000, Abcom, USA). The primary proteins were detected by using horseradish peroxidase-conjugated secondary antibodies (Abcam, USA), and the immune complexes were finally developed by using the enhanced chemiluminescence Plus kit (Amersham, Freiburg, Germany).

### 2.6. Real-Time PCR (RT-PCR) Analysis

Total RNA was extracted from renal tissues using Trizol according to the manufacturer's instructions (Takara, Japan). We obtained complementary DNA (cDNA) by reverse transcribing four micrograms of total RNA by using the PrimeScript RT Master Mix (Takara, Japan) as instructed. Real-time PCR amplifications were performed by using qPCR technique of SybrGreen assay on the ABI 7500 system (Applied Biosystems, USA). PCR primers (Takara, Japan) for all analyzed genes are as follows: tumor necrosis factor-*α* (TNF-*α*), amplicon size 122 bp, forward, 5′-GTG GAA CTG GCA GAA GAG GC-3′ and reverse, 5′-AGA CAG AAG AGC GTG GTG GC-3′; interleukin-1*β* (IL-1*β*), amplicon size 230 bp, forward, 5′-GCC CAT CCT CTG TGA CTC AT-3′ and reverse, 5′-AGG CCA CAG GTA TTT TGT CG-3′; COX-2, amplicon size 121 bp, sense: 5′-CCT GGT CTG ATG ATG TAT GC-3′; antisense: 5′-GTA TGA GTC TGC TGG TTT GG-3′; iNOS, amplicon size 108 bp, forward, 5′-TCC ATG ACT CCC AGC ACA-3′ and reverse, 5′-CCA TCT CCT GCA TTT CTT CC-3′; GAPDH, amplicon size 211 bp, forward, 5′-CAT CAA CGG GAA GCC CAT C-3′ and reverse, 5′-CTC GTG GTT CAC ACC CAT C-3′. PCR conditions were as follows: 94°C for 5 min; 35 cycles at 94°C for 40 s, 58°C for 40 s, and 72°C for 60 s; final elongation at 72°C for 10 min. The relative expression levels were calculated using the 2^−ΔΔCt^ method as reported.

### 2.7. Oxygen-Glucose Deprivation (OGD)

The human proximal tubular epithelial cells, HK-2 (ATCC-CRL-2190, Manassas, VA), were seeded in high glucose DMEM (Hyclone, USA) containing 10% fetal bovine serum (FBS; Gibco, USA) and incubated in humidified cell culture incubator containing gas mixture composed of 21% O_2_, 74% N_2_, and 5% CO_2_ at 37°C for 48 h. OGD followed by reoxygenation (OGD/R) was used to simulate an* in vitro* model of I/R injury [16]. Specifically, the cells in OGD group were incubated in glucose-free DMEM (Gibco, USA) without serum and placed in a hypoxic chamber (Billups-Rothenberg, USA) filled with anoxic gas mixture (95% N_2_/5% CO_2_) for 6 h. Meanwhile, cells in normal control were incubated in high glucose DMEM supplemented with 10% FBS and placed in a normoxic incubator. At the end of OGD, the plates were taken out from the hypoxic chamber, cells were transferred to high glucose DMEM containing 10% FBS, and they continued to incubate for 24 h under normoxic conditions to generate reoxygenation. In some groups, polydatin (10, 20, and 40 *μ*M) was continuously applied for 30 min before OGD to the end of reoxygenation. To determine the involvement of PI3K/Akt pathway, the PI3K/Akt inhibitor and wortmannin (1 *μ*M) were continuously applied for 30 min before OGD to the end of reoxygenation. In studies* in vitro* polydatin and wortmannin were first dissolved in DMSO (Sigma) and then diluted by DMEM with a final concentration of 1‰ (vol/vol) DMSO for cell treatment.

### 2.8. Cell Apoptosis Assay

We performed apoptosis assays by using an Annexin V-fluorescein isothiocyanate (FITC) apoptosis detection kit (catalog number 556419; BD Pharmingen) in accordance with the manufacturer's instructions. Briefly, HK-2 cells in the dish (10^5^ cells/well) were collected and then resuspended in binding buffer. Annexin V-FITC and PI were added into the single-cell suspension, which was then incubated in dark place for 15 min. Finally, cells were analyzed by a FACSCalibur flow cytometer (Becton Dickinson, BD Biosciences, USA).

### 2.9. Enzyme Linked Immunosorbent Assay (ELISA)

The levels of TNF-*α*, IL-1*β*, prostaglandin E-2 (PGE-2), and nitric oxide (NO) in renal tissue homogenate were measured by ELISA using a commercially available ELISA kit (R&D Systems, USA) referring to the manufacturer's recommendation. And the quantification of all these factors was implemented by using BCA protein assay reagent (Pierce, USA). Optical density values at 450 nm were measured with wavelength correction set to 570 nm. All standards and samples were measured in duplicate.

### 2.10. Measurement of Renal Oxidative Indexes

Renal tissue samples were weighed and homogenized (1 : 10, w/v) in 50 mmol/L phosphate buffer (PH 7.4) in an ice-bath and centrifuged at 1500 g for 20 min at 4°C. The supernatant was used to measure the activity of malondialdehyde (MDA), superoxide dismutase (SOD), glutathione transferase (GST), glutathione peroxidase (GPx), catalase (CAT), and the content of glutathione (GSH) that followed the commercial kit instructions by using a spectrophotometer (Spectrophotometer DU640, Beckman Coulter, Fullerton, CA) with the associated detection kits (Jiancheng, Nanjing, China). All of the levels are expressed as U/mg protein or nmol/mg protein, respectively.

### 2.11. Statistical Analysis

All of the values in the present study were expressed as means ± SD. Differences between data means were compared by use of analysis of variance (ANOVA) or Student's *t*-test by the SPSS statistical software package (SPSS, Inc., Chicago, IL, USA). A threshold of statistical significance was set at *P* < 0.05 for all analyses.

## 3. Results

### 3.1. Polydatin Improved the Renal Function in Renal I/R Injury Mice

First, we detected whether polydatin can improve the renal function of mice after I/R injury. For this purpose, BUN and Scr levels were examined at 12 h, 1 d, 3 d, 5 d, and 7 d after I/R injury in mice with different doses of polydatin administration, respectively. Compared with sham group, the renal functions of mice in vehicle group and polydatin groups were all worsened significantly, which suggested that the renal I/R models were successfully established in the present study (Figures 1(b) and 1(c)). The impaired renal function in mice of vehicle group self-recovered significantly at 7 days after I/R operation. However, compared with vehicle (saline) group, the administration of all three doses (10, 20, and 40 mg/kg) of polydatin significantly improved the impaired renal function of mice after I/R injury, but, respectively, at day 5 and 3 after I/R. These results suggested that polydatin can accelerate the recovery of renal function in mice after I/R injury in dose dependent manner (Figures 1(b) and 1(c)).

Histological examinations including HSK (Figure 1(d)), PCNA (Figure 1(e)), and TUNEL staining (Figure 1(f)) were evaluated at 12 h, 1 d, 3 d, 5 d, and 7 d after I/R. As expected, compared with control kidneys from saline-treated mice, polydatin reduced HSK, increased number of PCNA-positive cells, and decreased number of apoptotic cells on TUNEL assay (Figures 1(d)–1(f)). In particular, at 24 h after I/R injury, the number of PCNA-positive cells in kidneys from mice in polydatin-H group was significantly increased compared with that in the groups of vehicle (+10.2-fold), polydatin-L (+7.8-fold), and polydatin-M (+5.8-fold). Meanwhile, the similar increasement was delayed and detected, respectively, at 3 d, 5 d, and 7 d after I/R in polydatin-M, polydatin-L, and vehicle groups (Figures 1(d)–1(f)). The increase in renal cell survival following I/R injury was confirmed by measure of apoptosis using TUNEL analysis, which showed that polydatin remarkably decreased cell apoptosis in kidneys of mice after I/R, especially the polydatin-H groups, compared with vehicle control at 1 d, 3 d, 5 d, and 7 d after I/R (Figures 1(d)–1(f)).

Given that the beneficial effect of polydatin-H was the most significant at 3 d after I/R in mice, so the subsequent experiments in this study were all performed following this treatment.

### 3.2. PI3K/Akt Pathway Participated in the Nephroprotective Effects of Polydatin

**Figure 2:**
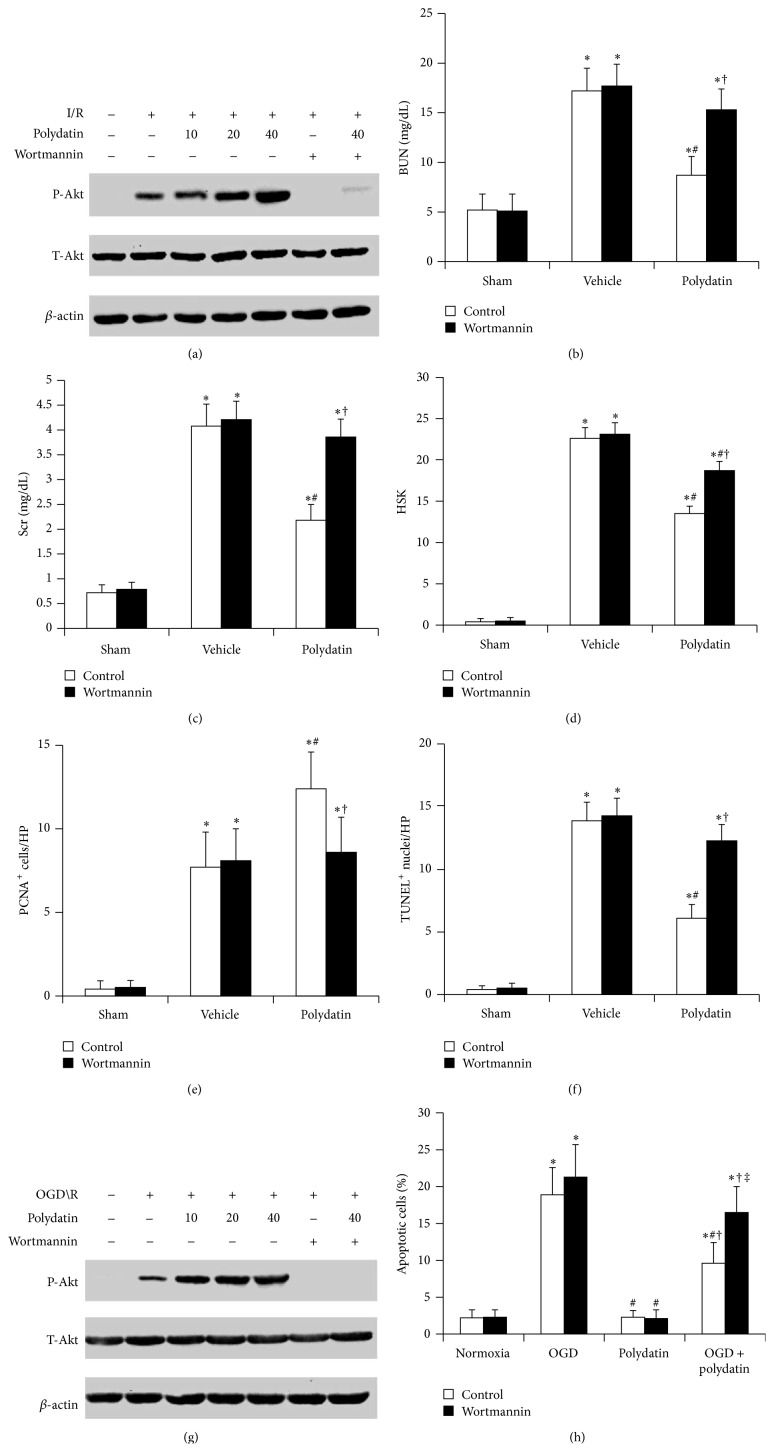
PI3K/Akt pathway participated in the nephroprotective effects of polydatin in renal I/R injury. (a) Western blot for P-Akt and T-Akt proteins in kidneys of mice at 3 d after I/R with or without polydatin and wortmannin treatment. *β*-actin was used as a control; (b) blood urea nitrogen (BUN), (c) serum creatinine (Scr), (d) histological score of kidney injuries (HSK), (e) renal PCNA expression, (f) renal TUNEL-apoptosis were detected in mice at 3 d after I/R with or without 40 mg/kg of polydatin and 1 mg/kg wortmannin treatment. ^*^ *P* < 0.05 versus sham; ^#^ *P* < 0.05 versus vehicle; ^†^ *P* < 0.05 versus control; (g) P-Akt and T-Akt protein levels in cells with or without OGD/R, in presence or absence of polydatin (10, 20, and 40 *μ*M) and wortmannin (1 *μ*M). (h)* In vitro* survival analysis of human proximal tubular epithelial HK-2 cells treated with or without polydatin (20 *μ*M) and wortmannin (1 *μ*M) in basal conditions and after OGD/R. Bar graph described from the FACS-based Annexin V/propidium iodide apoptosis assay. ^*^ *P* < 0.05 versus normoxia; ^#^ *P* < 0.05 versus OGD; ^†^ *P* < 0.05 versus polydatin; ^‡^ *P* < 0.05 versus control.

To validate the association between Akt signaling and nephroprotective effect of polydatin, we detected the activation of Akt. Compared with sham group, the level of p-Akt increased at 3 d after I/R; polydatin dose dependently further elevated the I/R-induced increase of p-Akt. Intraperitoneal injection of the inhibitor of PI3K/Akt, wortmannin, significantly blocked the polydatin-elevated phosphorylation of Akt (Figure 2(a)). In mice of sham group, the renal function after operation was not affected by wortmannin, suggesting that wortmannin had no apparent renal toxicity (data not shown). However, wortmannin significantly reversed the beneficial effect of polydatin in decreasing the levels of BUN and Scr in renal I/R injury mice (Figures 2(b) and 2(c)). Meanwhile, compared with polydatin-H group, kidneys from mice treated with both polydatin-H and wortmannin had significantly increased HSK and the percentage of apoptotic cells on TUNEL assay and reduced number of PCNA-positive cells (Figures 2(d)–2(f)).

To further confirm the results* in vivo*,* in vitro* renal I/R injury models were simulated. The results of western blot showed that the phosphorylation of Akt was activated by OGD/R and further elevated by polydatin dose dependently, but it was counteracted in the presence of wortmannin (Figure 2(g)). The results of apoptosis assays showed that, compared with normal cultured cells, OGD/R notably increased the apoptosis of HK-2 cells, which was obviously suppressed by 20 *μ*M of polydatin (Figure 2(h)). Wortmannin did not increase the cell apoptosis under normoxic conditions which revealed that wortmannin had no apparent cytotoxicity. However, wortmannin not only further increased the OGD/R-induced apoptosis, but also obviously blocked the protective effects of polydatin on HK-2 cells (Figure 2(h)). In short, these results suggested that the nephroprotective effects of polydatin were associated with the PI3K/Akt signaling pathway.

### 3.3. PI3K/Akt Pathway Is Involved in Polydatin-Attenuated Expression of the Proinflammatory Factors in Renal I/R Injury

**Figure 3:**
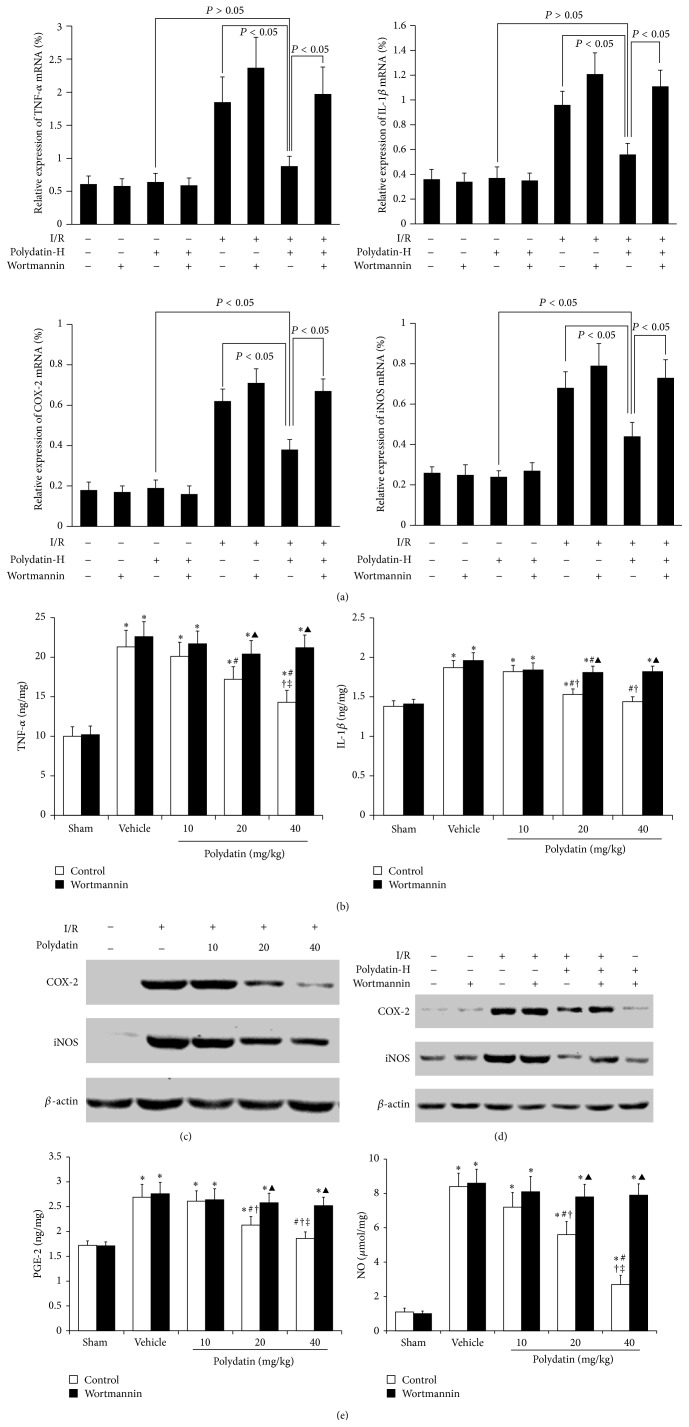
PI3K/Akt pathway was involved in polydatin-attenuated expression of the proinflammatory factors. (a) RT-PCR was used for the analysis of TNF-*α*, IL-1*β*, COX-2, and iNOS mRNA levels in kidneys of mice with or without polydatin-H (40 mg/kg) and wortmannin (1 mg/kg) at 3 d after I/R. GAPDH was used as a control. (b) ELISA was used to detect the protein levels of TNF-*α* and IL-1*β* in renal tissues in mice at 3 d after I/R administrated with different doses of polydatin, with or without wortmannin (1 mg/kg), intraperitoneally. (c) Western blot analysis assessed the expression of COX-2 and iNOS in kidneys of mice at 3 d after I/R with different doses of polydatin treatment. *β*-actin was used as a control. (d) Western blot analysis was used to detect the expression of COX-2 and iNOS in kidneys of mice at 3 d after I/R with polydatin-H treatment in presence or absence of wortmannin (1 mg/kg). *β*-actin was used as a control. (e) ELISA was used to detect the levels of PGE-2 and NO in renal tissues in mice at 3 d after I/R administrated with different doses of polydatin, with or without wortmannin (1 mg/kg), intraperitoneally. ^*^ *P* < 0.05 versus sham; ^#^ *P* < 0.05 versus vehicle; ^†^ *P* < 0.05 versus polydatin (10 mg/kg); ^‡^ *P* < 0.05 versus polydatin (20 mg/kg). ^▲^ *P* < 0.05 versus control.

To evaluate the potential anti-inflammation effects of polydatin in renal I/R injury, we assessed the expression of TNF-*α*, IL-1*β*, COX-2, and iNOS in the kidneys from mice at 3 d after I/R. RT-PCR showed that these cytokines were extensively expressed in kidneys from mice after I/R injury, but only mildly expressed in kidneys from mice with polydatin treatment. However, wortmannin significantly increased the polydatin-attenuated expression of the proinflammatory factors induced by I/R (Figure 3(a)). ELISA analysis showed that the expression of TNF-*α* and IL-1*β* was increased at 72 h after I/R injury, which was decreased by polydatin in dose dependent manner (Figure 3(b)). Western blot analysis showed that the expression of COX-2 and iNOS was significantly increased in kidneys at 72 h after I/R injury, which was dose dependently decreased by polydatin (Figure 3(c)). These results suggested the beneficial effect of polydatin on ameliorating the inflammation in renal I/R injury mouse model.

Meanwhile, the intraperitoneal injection of wortmannin obviously abolished the polydatin-induced decreased expression of TNF-*α*, IL-1*β*, COX-2, and iNOS (Figures 3(b) and 3(d)). Additionally, we also detected PGE-2 and NO and the downstream factors of COX-2 and iNOS, respectively. Similarly, the levels of PGE-2 and NO were significantly increased by I/R, obviously suppressed by polydatin, and went up again when treating mice with wortmannin (Figure 3(e)). These results indicated that PI3K/Akt pathway is involved in the anti-inflammation effect of polydatin in renal I/R injury mice.

### 3.4. PI3K/Akt Pathway Was Associated with Polydatin-Attenuated Oxidative Stress in Renal I/R Injury

**Figure 4:**
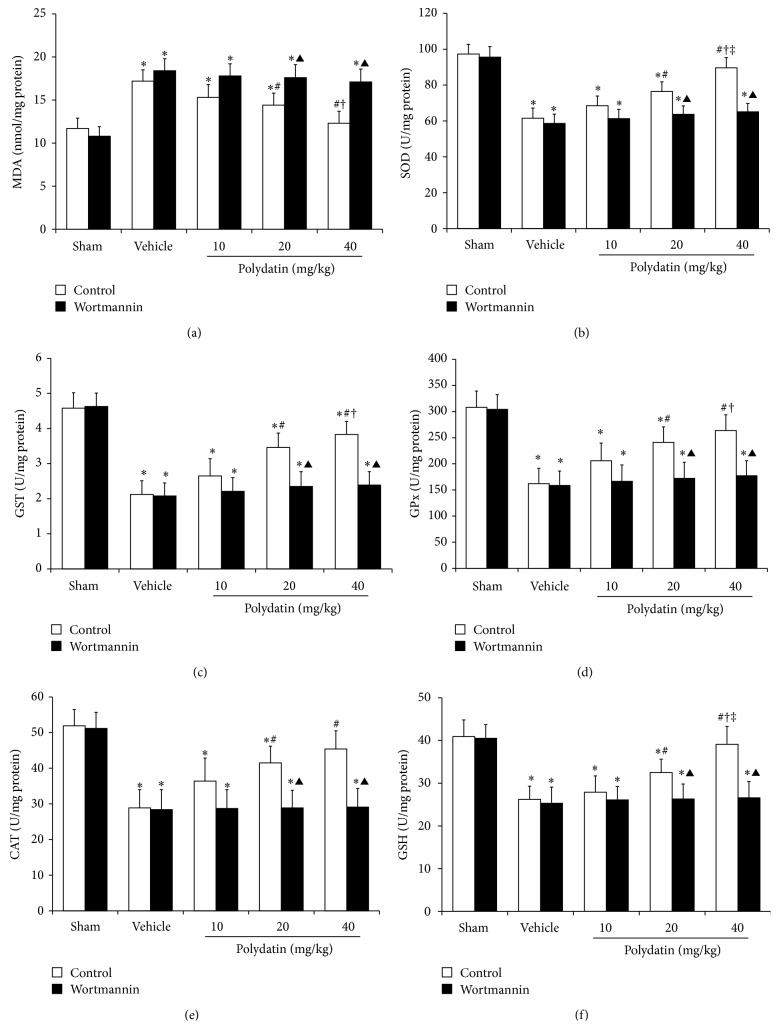
PI3K/Akt pathway was associated with polydatin-attenuated oxidative stress in renal I/R injury. Measurement of MDA (a), SOD (b), GST (c), GPx (d), CAT (e), and GSH (f) was performed on mice treated with different doses of polydatin and with or without wortmannin (1 mg/kg) at 3 d after I/R. ^*^ *P* < 0.05 versus sham; ^#^ *P* < 0.05 versus vehicle; ^†^ *P* < 0.05 versus polydatin (10 mg/kg); ^‡^ *P* < 0.05 versus polydatin (20 mg/kg). ^▲^ *P* < 0.05 versus control.

To validate the potential effect of polydatin on antioxidative stress in renal I/R injury, we detected the contents of MDA and GSH and the activity of four antioxidases (SOD, GST, GPx, and CAT) in kidneys, respectively. Compared with sham group, the MDA content was significantly increased in I/R injury mice and was reversed in polydatin-M and polydatin-H groups but not in polydatin-L group (Figure 4(a)), while the activities of the four antioxidases were all significantly decreased in the kidneys of I/R injury mice, and polydatin elevated the activity that was decreased by I/R (Figures 4(b)–4(e)). Compared with vehicle group, the activities of SOD, GST, GPx, and CAT were all significantly increased in polydatin groups except polydatin-L group (Figures 4(b)–4(e)). Compared with sham group, the level of GSH decreased in I/R injury mice. Compared with vehicle group, the GSH content was elevated in polydatin-M and polydatin-H group, but not in polydatin-L group (Figure 4(f)). All measurements mentioned above had no significant statistic difference between sham group and polydatin-H group. These results suggested the antioxidative stress effect of polydatin in alleviating renal I/R injury. However, wortmannin significantly abolished the effect of polydatin on decreasing MDA content, increasing the activity of SOD, GST, GPx, and CAT, and elevating the level of GSH (Figure 4), which suggested that PI3K/Akt pathway was associated with polydatin-attenuated oxidative stress in renal I/R injury.

## 4. Discussion

Polydatin is an active stilbene compound isolated from the roots of* Polygonum cuspidatum* Sieb. et Zucc. and has been manifested to possess antioxidative and anti-inflammatory activities [5, 9, 17]. We and other authors have demonstrated the therapeutical effects of polydatin on I/R-induced injury in multiple organs including heart and brain [4, 5, 18–20]. It has been identified that polydatin also has nephroprotective effects in diabetes and urate nephropathy through prevention of oxidative stress and inflammation [6–9, 21]. In the present study, we further investigated the potential therapeutic effects and mechanism of polydatin on renal I/R injury. Our results showed that the administration of polydatin significantly improved the renal function, accelerated the mitogenic response, and reduced HSK and cell apoptosis in renal I/R injury models, suggesting the beneficial effect of polydatin against renal I/R injury.

In fact, renal I/R injury always induced the excessive generation of proinflammatory cytokines in kidneys, which resulted in leukocyte infiltration and tissue damage [22, 23]. TNF-*α* and IL-1*β* are two important proinflammatory mediators in renal I/R injury, which produce a number of injurious changes in proximal tubular epithelial cells [22–26]. Loss of TNF-*α*, either through using neutralization antibody of TNF-*α* blockade or knockout mice of TNF-*α*, resulted in significantly alleviated tissue injury and elevated function in kidneys after renal ischemia, while the transgenic mice with TNF-*α* overexpression had more pronounced susceptibility to acute kidney injury induced by I/R than that in mice of wild type [27]. There were also enormous studies that have demonstrated that decreasing IL-1*β* was associated with improved renal function in renal I/R injury models [28–30]. Therefore, we examined the levels of TNF-*α* and IL-1*β* to determine whether they were associated with the mechanism of polydatin in treating renal I/R injury diseases. The results showed that polydatin significantly decreased I/R-induced TNF-*α* and IL-1*β* levels in kidneys. These data suggested that the positive effects of polydatin on improving renal function in mice after I/R injury might be achieved by inhibiting the proinflammatory cytokines of TNF-*α* and IL-1*β*.

With the exception of proinflammatory cytokines of TNF-*α* and IL-1*β*, COX-2 and iNOS, two enzymes associated with inflammation, were also correlated with the pathogenesis of I/R injury. COX-2, an inducible enzyme, plays crucial roles in regulating the inflammatory response and oxidative stress in I/R injury [31]. Selective or nonselective inhibition of COX-2 with either rofecoxib or indomethacin ameliorated renal tissue damage induced by I/R injury [32]. Another study has shown that continuous intrarenal infusion of parecoxib (40 mg per pig) improved renal function in pigs with the operation of suprarenal aortic cross clamping [33]. These results of previous studies suggested that inhibition of COX-2 has the potential to improve renal function in renal I/R injury. iNOS which was highly expressed after renal I/R injury mediated the generation of NO [34, 35]. High activity of iNOS aggravated the damage of kidneys in renal I/R injury, and selective inhibition of iNOS significantly improved the renal function in rats with I/R [36–38]. Based on the results of the studies above, we detected the expressions of COX-2 and iNOS protein and the contents of PEG_2_ and NO, downstream factors of COX-2 and iNOS, in the kidneys after renal I/R injury. Our results showed that, compared with mice in vehicle group, polydatin notably inhibited the expressions of COX-2 and iNOS protein which were upregulated by I/R in renal I/R mice, dose dependently. The results were in line with the improved renal function induced by polydatin (Figure 1), indicating that the protective effects of polydatin on acute renal I/R injury might be by inhibiting the expression of the potential inflammatory mediators of COX-2 and iNOS and decreasing the generation of the downstream factors of them, PEG-2 and NO.

There are compelling evidences that oxidative stress is particularly involved in the pathogenesis of renal I/R injury [35]. Briefly, I/R has been proposed to have the potential to promote oxidative stress, which in turn can promote I/R injury [25]. The elevation of oxidative molecules and the reduction of antioxidant substance can aggravate I/R injury. It has been demonstrated that polydatin was a prominent antioxidant and exhibited cardioprotective effects in myocardial I/R injury rat models via increasing SOD activity and decreasing MDA content [4, 5]. The results in our experiment substantiated that polydatin significantly elevated the activity of SOD, GST, GPx, and CAT, increased GSH level, and decreased the MDA content in kidneys of I/R injury mice, which indicated the prominently antioxidative properties of polydatin and was in line with the data previously reported by Chen et al. in urate nephropathic mice [9].

The PI3K/Akt pathway was originally recognized to play a crucial role in regulating the growth and survival of cells, which nowadays has been rediscovered to be implicated in the protection of brain, myocardium, lung, liver, and kidney against I/R injury by regulating oxidative stress and inflammatory response [39–44]. Recently it has been demonstrated that polydatin exerted hepatoprotective effect in rats fed with high-fat diet [45] and regulated glucose and lipid metabolism in diabetic models [46] through upregulating the phosphorylation of Akt in liver, and polydatin also exhibited antitumor activity [47] through downregulating the phosphorylation of Akt in human nasopharyngeal carcinoma CNE cells. These results suggested that Akt signaling pathway was a potential therapeutic target of polydatin in treating various diseases. Therefore, in the present study, we assessed whether PI3K/Akt pathway was associated with the nephroprotective effects of polydatin in renal I/R injury models. First, we identified that the phosphorylation of Akt was activated in renal tissues by I/R and further increased by polydatin in dose dependent manner. However, the polydatin-induced increase of phosphorylation of Akt was significantly decreased by the specific PI3K/Akt inhibitor, wortmannin, suggesting the positive role of polydatin on the activation of Akt in renal I/R injury. Importantly, when blocking the phosphorylation of Akt by intraperitoneal injection of wortmannin, the beneficial effect of polydatin on the regeneration of renal tissues was also abolished. And wortmannin remarkably counteracted the polydatin-attenuated levels of proinflammatory factors and oxidative stress in kidneys of I/R injury. These results suggested that PI3K/Akt pathway, at least partly, was involved in the nephroprotective effects of polydatin in renal I/R injury. Of course, the potential downstream functional molecules mediated by PI3K/Akt signaling pathway to take part in polydatin's actions still remain to be further investigated.

## 5. Conclusions

In this study, we identified for the first time that, in acute renal I/R injury models, the administration of polydatin significantly improved the renal function, accelerated the mitogenic response, reduced cell apoptosis, strongly suppressed the I/R-induced upregulation of the expression of TNF-*α*, IL-1*β*, COX-2, PGE-2, iNOS, and NO, and dramatically decreased contents of MDA, but increased the activity of SOD, GPx, GST, CAT, and the level of GSH. However, all these beneficial effects of polydatin were counteracted when we inhibited PI3K/Akt pathway with its specific inhibitor, wortmannin. These findings taken together elucidated that polydatin exhibited prominent nephroprotective effects against renal I/R injury, at least in part, through PI3K/Akt-dependent phosphorylation. In conclusion, our data support that polydatin is promising to be a good drug for prevention and treatment of I/R-induced renal injury in the clinical practice.
